# Transformation of lymphoid interstitial pneumonia (LIP) into malignant lymphoma in patients with Sjogren's syndrome: a case report and literature review

**DOI:** 10.1186/s13019-022-01826-6

**Published:** 2022-04-15

**Authors:** Chengyuan Zhu, Jibo Hu, Jun Wu, Lingxiao Cheng

**Affiliations:** 1 Department of Medical Imaging and Nuclear Medicine, Shaoxing University School of Medicine, Shaoxing, 312000 Zhejiang China; 2 Department of Medical Imaging Center, Sir Run Run Shaw Hospital, Zhejiang University School of Medicine, Hangzhou, 310016 Zhejiang China; 3 Department of Radiology, The Fourth Affiliated Hospital, Zhejiang University School of Medicine, Yiwu, 322001 Zhejiang China; 4 Health Promotion Center, Sir Run Run Shaw Hospital, Zhejiang University School of Medicine, Hangzhou, 310016 Zhejiang China

**Keywords:** Lymphocytic interstitial pneumonia, Sjogren's syndrome, Lymphoma, Malignant transformation, Case report

## Abstract

**Background:**

Lymphoid interstitial pneumonia (LIP) is a very rare disease and its malignant transformation is even more rare. LIP is easily misdiagnosed by clinicians and radiologists.

**Case presentation:**

The medical record of a 64-year-old female with Sjogren's syndrome was reviewed. The clinical and pathological data along with chest CT images were obtained. The literature related to the transformation was reviewed. There were no specific clinical manifestations of LIP and its transformation into malignant lymphoma in the patient. The chest CT mainly displayed multiple cystic foci, with multiple nodules and ground-glass shadows in both lungs.

**Conclusions:**

Malignant transformation to lymphoma is suspected with findings of large nodules (> 11 mm) or their sizes doubly increased, pleural effusion and alveolar consolidation.

## Introduction

Lymphocytic interstitial pneumonia (LIP) is a benign parenchymal polyclonal proliferative disorder in the lung characterized by diffuse lymphocytic infiltration admixed with plasma cells and other cellular elements. It has a low incidence and is easily misdiagnosed or missed in clinical evaluation. The mortality rate for patients with LIP is 33–50% within 5 years of diagnosis. Reports indicate that about 5% of patients developed low grade malignant B cell lymphoma [1–3]. Herein, we reported a case of LIP that was initially misdiagnosed and which later transformed into malignant pulmonary lymphoma.

## Case presentation

Due to aggravated cough and expectoration with fever, chest tightness and shortness of breath, a 64-year-old female was admitted to our hospital four days ago. She had been diagnosed with xerostomia and xerophthalmia over 20 years.

**Fig. 1:**
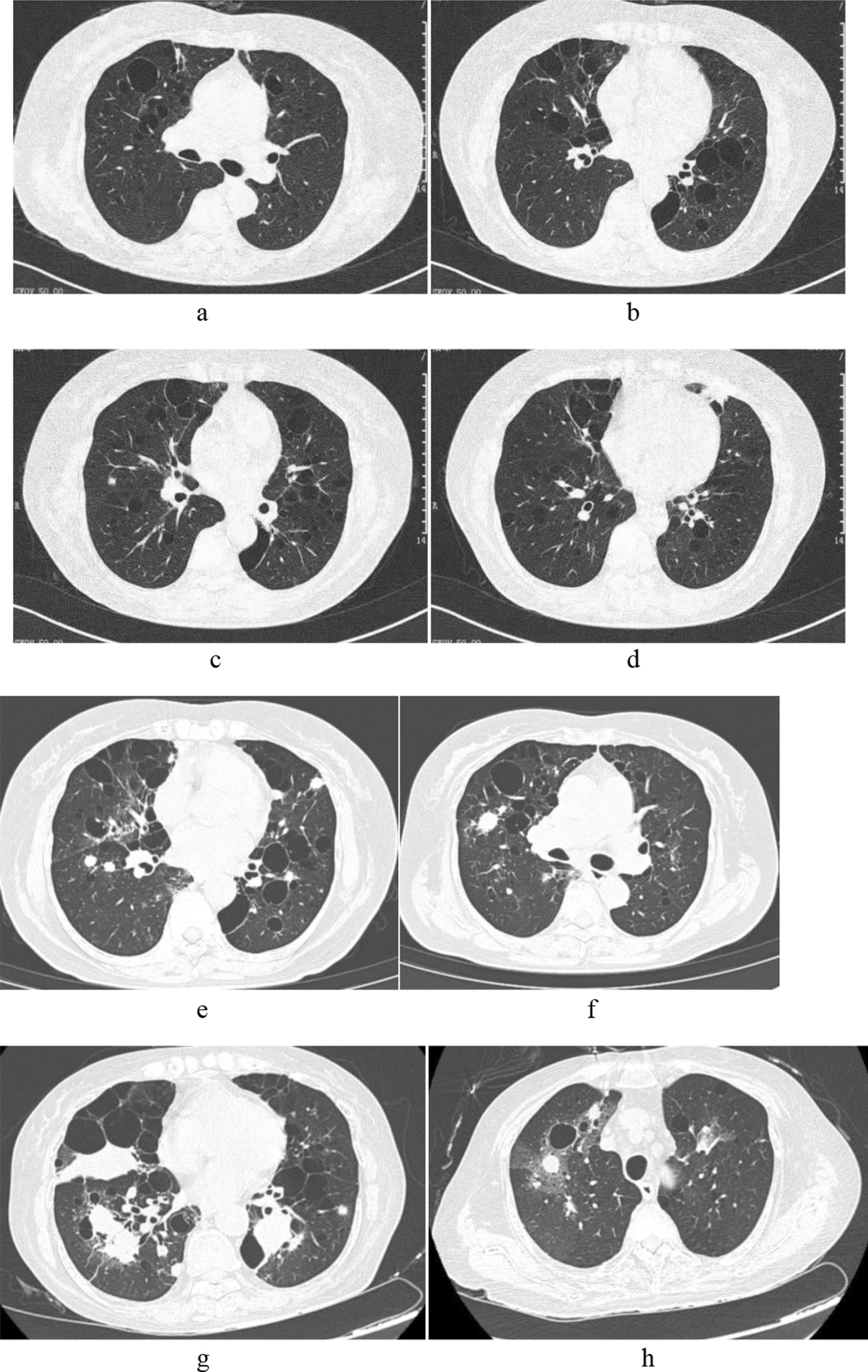
**a**, **b** Chest CT three years ago showed scattered, various-sized roundish radiolucencies, and scattered striped and blurred shadows in two lungs. **c**, **d** Chest CT two years ago showed new patchy opacities found in the right lung and lingual lobe, multiple cystic foci, and nodules and opacities in both lungs. **e**, **f** Chest CT eight months before admission showed multiple cystic foci in both lungs, diffuse multiple nodules and patches of varied sizes in both lungs. **g**, **h** Chest enhanced CT 4 days after admission showed that patchy lesions and nodules in both lungs were more advanced than before, and some of the lesions were surrounded by patchy ground glass opacities

Emission Computed Tomography (ECT) examination revealed decreased functions of bilateral parotid and submandibular glands. A chest CT three years ago showed scattered, various-sized and roundish radiolucencies, striped or hazy opacities in both lungs and multiple small nodules in the right lung (Fig. 1a, b). Another chest CT one year later revealed new patchy opacities in the right lung and lingual lobe, scattered various-sized and roundish radiolucencies, multiple small nodules in the right lung which were similar to those in the previous CT image; the striped or hazy opacities seen previously were still visible in both lungs (Fig. 1c, d).

**Fig. 2:**
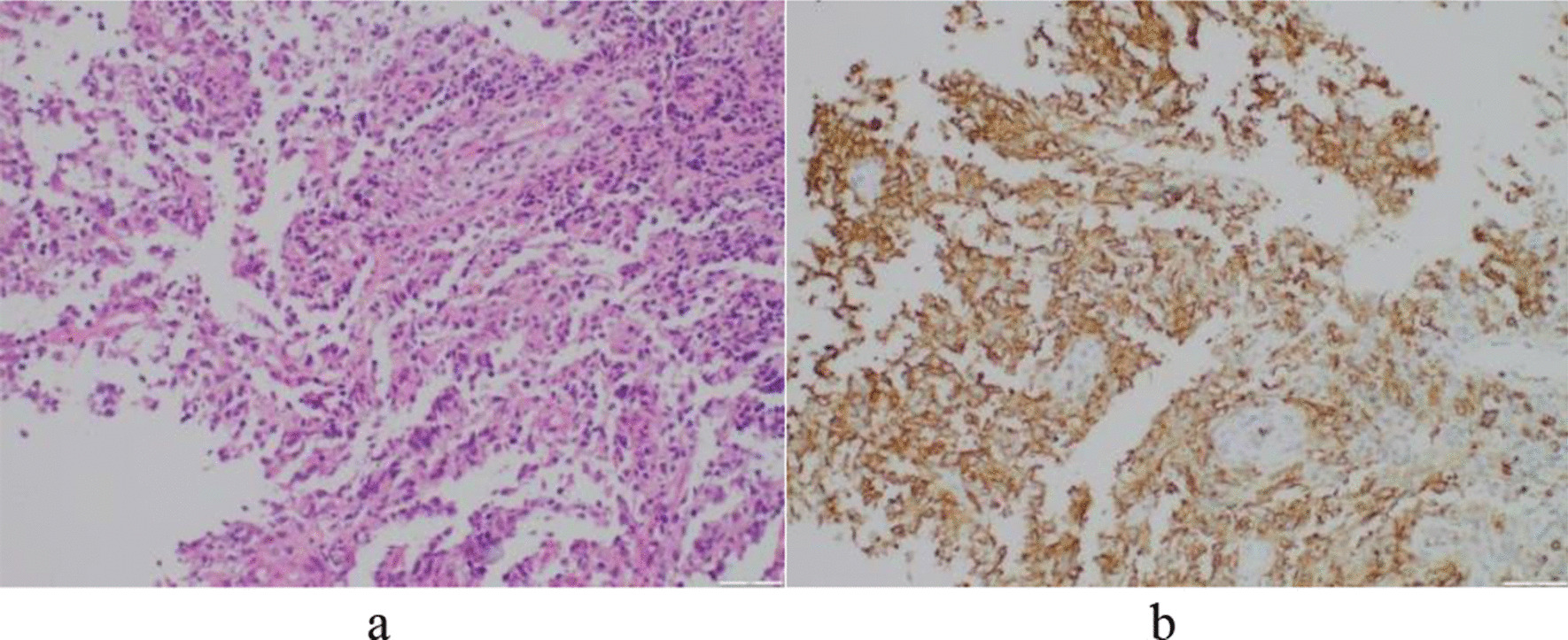
**a** Pulmonary interstitial infiltration by a large number of lymphocytes (×200). **b** Immunohistochemistry showed positive staining for CD20 on lymphocytes (× 200)

The patient was admitted to our hospital one year and 4 months later due to recurrent cough, expectoration and low fever. The chest CT revealed multiple cysts with multiple patchy and nodular opacities in both lungs (Fig. 1e, f). Results of antinuclear antibody profiles and vasculitis series were: SSA (RO-52) positive (+), SSA antibody positive (+), ESR 87 mm/hr, immunoglobulins: IgA 4.29 g/L, IgG 20.3 g/L and IgM 0.68 g/L. Blood gas analysis: pH 7.39, pCO2 39.5 mmHg, pO2 68.4 mmHg and SaO2 93.1%. Pathological report on (right lung biopsy): Chronic inflammation of lung tissue with diffuse distribution of lymphocytes in focal areas (Fig. 2a). Diagnosis: lymphoma could not be excluded. Preliminary clinical diagnosis: (1) Sjogren's syndrome, lymphocytic interstitial pneumonia first consideration; (2) malignant lymphoma to be excluded; (3) pulmonary infection. The patient received anti-inflammatory treatment and was discharged after symptoms were improved.

After discharge, the patient continued to experience constant cough, expectoration and fever even after repeated anti-inflammatory treatments. A left lung biopsy was performed 4 months later during her inpatient treatment in another hospital. The pathological report indicated chronic suppurative inflammation with massive necrosis and cellulitis.

The patient’s chest enhancement CT after this admission displayed obviously more patchy and nodular foci in both lungs compared to previous CT images. Some of the lesions were surrounded with ground glass shadows (Fig. 1g, h). The diagnosis was: malignant tumor to be excluded. Auxiliary routine blood examinations showed white blood cell count 21.8 × 10^9^/L and absolute neutrophil count 21.14 × 10^9^/L.

Pathological results from our hospital (right lung biopsy) and another hospital showed small areas of proliferating inflammatory fibrous tissue and small numbers of heterotypic cells within the necrotic tissue. Malignancy could not be excluded.

Immunohistochemistry showed: CD20 (+) (Fig. 2b), Ki-67 (high-value-added activity), BCL-6 (+), CD21 (+). Based on history and pathological results, the final diagnosis was Sjogren's syndrome, malignant transformation of LIP into diffuse large B-cell lymphoma. Subsequently, chemotherapy with a reduced-dose regimen of rituximab and Chop (R-miniCHOP) was administered eight times. Following treatment, PET/CT showed complete remission of lymphoma, but the patient was at high risk of recurrence and is under active follow-up.

## Discussion

LIP was first described by Liebow and Carrington in 1966. Both the American Thoracic Society and the European Respiratory Society classified LIP as idiopathic interstitial pneumonia in 2002 [1]. LIP is a disease of slow progression. Patients with the disease usually have a long lifespan. Most patients are females and middle-aged or elderly, with an average age of initial diagnosis of 52–56 years. LIP can also occur in children, especially in those with hypogammaglobulinemia and AIDS. Clinical manifestations of LIP are nonspecific. The most common symptoms are dry cough and progressive dyspnea. Other symptoms include wasting, fever, joint and chest pain, and occasional hemoptysis [2]. Velcro rales can be heard at the bottoms of both lungs. Clubbing fingers may also be present but are rare. The patient in our case study presented with repeated low fever and insensitivity to anti-inflammatory treatments after transformation of LIP to lymphoma. More studies are needed to determine whether repeated low fever and poor response to anti-inflammatory treatments are clinically valuable in diagnosis of malignant lymphoma transformed from LIP.

Primary LIP is relatively rare. Most cases are secondary, and a few are idiopathic. The exact etiology is not clear. Studies have shown that this disease is often associated with autoimmune diseases and viral infections. Epstein-Barr virus DNA can be detected in both children and adults with LIP in some cases and has been considered to be causative [1]. HIV infection is also associated with LIP. It occurs in 16–50% of children infected with HIV and in less than 5% of adult HIV patients [3]. LIP is relatively common in patients with autoimmune diseases, including Sjogren's syndrome, rheumatoid arthritis, systemic lupus erythematosus, Hishimoto's thyroiditis, and polymyositis. Among them, Sjogren's syndrome is the most common [3, 4]. Approximately 25% of LIP is associated with Sjogren's syndrome and approximately 1% of patients with Sjogren's syndrome will develop LIP during the course of the disease [1]. It is notable that our patient had a history of Sjogren's syndrome for many years.

Approximately 80% of LIP patients may have abnormal serum proteins. The most common are polyclonal gamma globulins, with IgM as the major type, and occasionally IgG [1, 3]. Hypogammaglobulinemia or monoclonal gammopathy may indicate concurrent lymphoma. Patients present with restrictive ventilatory dysfunction, reduced diffusing capacity, and variable degrees of hypoxemia. The results of pulmonary function tests and blood gas analysis reflect the severity of the disease. Bronchoalveolar lavage (BAL) is also a valuable indicator in the diagnosis of the disease. The increase in lymphocytes, CD3 cells and polyclonal CD20 cells in BAL is generally indicative of LIP [3, 5]. However, the final diagnosis of LIP depends on lung biopsy, which is pathologically characterized by diffuse lymphocytic infiltration in the pulmonary interstitium. It often displays lymphoid follicles with mainly lymphocytes (T cells and multicellular B cells), plasma cells, immunoblastic cells and histiocytes. The lesion is accompanied by thickening of the alveolar and interlobular compartments. Sometimes, it also involves peribronchial, but usually with mild lesions. Immunohistochemical studies are needed to determine the polyclonal nature of lymphocytes and to differentiate LIP from lymphoma.

In X-rays, LIP shows no specificity or abnormality, although it may show reticular or nodular shadows in the lower field of both lungs. High resolution CTs (HRCTs) show ground glass opacities, central lobular and subpleural nodules with unclear boundary accompanied by thickening of interlobular septum and bronchial blood bundle, more common in the lower lobes [6, 7]. Thin-walled cystic cavities are found in 68–82% of patients. These are found around blood vessels and range in size from 1 to 30 mm; a maximum size of 10 cm has been reported [8]. If not absorbed, appearance of central lobular nodules could progress to formation of cystic cavities. The pathophysiological mechanisms of formation of the thin-walled cystic cavities are that the infiltrated lymphocytes around the bronchioles progressively obstruct and dilatate the airway and cystic dilatation due to a flap effect [9]. Mediastinal lymphadenopathy is common in pediatric patients and in patients with Sjogren's syndrome. Among them, ground glass and scattered cystic shadows have certain diagnostic values. In this study, the patient has multiple parenchymal cystic cavities. LIP needs to be excluded when the parenchymal cystic cavities are observed. According to literature reports, nearly 5% of LIP patients develop low grade malignant B cell lymphoma. Early diagnosis of transformed lymphoma from LIP is very important for the whole treatment process. Imaging examination is an important means in the diagnosis of the disease. Pleural effusion and alveolar consolidation in LIP are rare and often suggest its transformation into malignancy, such as lymphoma. Large nodules (> 11 mm) or multiplicative nodules also indicate suspicious concurrent lymphoma. Case reports have shown that nodules larger than 11 mm exhibited increased metabolic activity on PET/CT in patients with LIP. The diagnostic value of PET/CT is limited, however, because both LIP with large nodules greater than 11 mm in diameter and primary lung lymphoma can show enhanced metabolic activity [10].

LIP must be distinguished from primary malignant lymphoma of the lung, nonspecific interstitial pneumonia (NSIP), pulmonary lymphangioleiomyomatosis (LAM), and pulmonary Langerhans cell histiocytosis (PLCH). Differentiation from primary malignant lymphoma of the lung: LIP lesion displays diffuse infiltration of lymphocytes, while primary malignant lymphoma is a localized lesion. The lymphocytes of LIP are polyclonal and those of primary malignant lymphoma are monoclonal. Immunohistochemistry and molecular gene rearrangement test are also helpful in differential diagnosis [3]. Differentiation from NSIP: The main pathological features of NSIP are diffuse distribution of pulmonary lesions, uniform with each other with no structural remodeling. The extent of inflammatory cell infiltration is less than that of LIP. Some alveolar walls remain uninvolved. The CT finding of extensive ground-glass abnormality and some reticular abnormality, with basal and peripheral predominance, is strongly suggestive of NSIP [11–13]. Differentiation from pulmonary LAM: The common manifestation of LAM is pneumothorax. LAM is characterized by proliferation of neoplastic smooth muscle cells (LAM cells) in the lung parenchyma. High-resolution CT demonstrates a large number of cystic lesions evenly distributed throughout the lung tissue with normal intermediate lung parenchyma [9, 14]. Differentiation from PLCH: Most patients with PLCH usually have a history of smoking. High resolution CT will show cystic changes, mainly distributed in the upper and middle lung fields, and no invasion of the costophrenic angle [9, 14].

In summary, there are no specific clinical manifestations for LIP and its transformed malignant lymphoma. The imaging findings are diverse. Ground glass opacities and small nodular and scattered cystic shadows in both lungs are suggestive of the diagnosis. During follow-up, suspected diagnosis of concurrent lymphoma needs to be raised in LIP patients at presence of large nodules (> 11 mm) or their sizes doubly increased, pleural effusion, and alveolar consolidation. Improvement in the understanding of malignant transformation of LIP to achieve early diagnosis is conducive to effective treatment and improved prognosis for patients. Due to the limited number of cases, the optimal treatment for such patients is difficult to determine. In general, given the malignant nature of lymphoma, chemotherapy is preferred.

## Data Availability

Not applicable.

## Abbreviations

LIP: Lymphoid interstitial pneumonia
CT: Computerized tomography
ECT: Emission Computed Tomography
PET/CT: Positron emission tomography/computed tomography
NSIP: Nonspecific interstitial pneumonia
LAM: Lymphangioleiomyomatosis
PLCH: Pulmonary Langerhans cell histiocytosis
